# The Reuniens Nucleus of the Thalamus Has an Essential Role in Coordinating Slow-Wave Activity between Neocortex and Hippocampus

**DOI:** 10.1523/ENEURO.0365-19.2019

**Published:** 2019-10-16

**Authors:** Brandon E. Hauer, Silvia Pagliardini, Clayton T. Dickson

**Affiliations:** 1 Neuroscience and Mental Health Institute, University of Alberta, Edmonton, Alberta T6G 2E9, Canada; 2 Department of Physiology, University of Alberta, Edmonton, Alberta T6G 2E9, Canada; 3 Department of Psychology, University of Alberta, Edmonton, Alberta T6G 2E9, Canada

**Keywords:** corticothalamic circuits, medial prefrontal cortex, memory, sleep, slow oscillation, synchronization

## Abstract

Sleep is a period of profound neural synchrony throughout the brain, a phenomenon involved in various physiological functions. The coordination between neocortex and hippocampus, in particular, appears to be critical for episodic memory, and, indeed, enhanced synchrony in this circuit is a hallmark of slow-wave sleep. However, it is unclear how this coordination is mediated. To this end, we examined the role of the thalamic nucleus reuniens (RE), a midline body with reciprocal connections to both prefrontal and hippocampal cortices. Using a combination of electrophysiological, optogenetic, and chemogenetic techniques in the urethane-anesthetized rat (a model of forebrain sleep activity), we directly assessed the role of the RE in mediating slow oscillatory synchrony. Using unit recording techniques, we confirmed that RE neurons showed slow rhythmic activity patterns during deactivated forebrain states that were coupled to ongoing slow oscillations. Optogenetic activation of RE neurons or their projection fibers in the cingulum bundle caused an evoked potential in hippocampus that was maximal at the level of stratum lacunosum-moleculare of CA1. A similar but longer-latency response could be evoked by stimulation of the medial prefrontal cortex that was then abolished by chemogenetic inhibition of the RE. Inactivation of the RE also severely reduced the coherence of the slow oscillation across cortical and hippocampal sites, suggesting that its activity is necessary to couple slow-wave activity across these regions. These results indicate an essential role of the RE in coordinating neocortico-hippocampal slow oscillatory activity, which may be fundamental for slow-wave sleep-related episodic memory consolidation.

## Significance Statement

Off-line reactivation of neural activity patterns occurring during previous waking periods might provide further activity-dependent solidification of the synaptic connections that would allow this neural information to be encoded more permanently. In other words, brain activity during sleep might benefit memory permanence. In this work, we show how two distant memory-related areas in the brain, the medial prefrontal cortex, and the hippocampus might coordinate their activity during slow-wave activity via an interposed thalamic structure, the nucleus reuniens. This circuit has already been suggested to play an important role during on-line memory processing; here, we show its potential relevance to off-line memory consolidation via its powerful ability to coordinate two episodic memory structures during slow-wave activity.

## Introduction

Although a lack of behavioral responsiveness during sleep suggests neural inactivity, the complicated and orchestrated dynamics of ongoing brain activity during this state entirely contradicts this assumption. Indeed, sleep is a period of profound and dynamic neuronal synchrony, and few activity patterns are better suited to coordinate widespread forebrain networks than the slow oscillation (SO; Cox et al., 2014). A major constituent of non-rapid eye movement (NREM) sleep, the SO is characterized by large-amplitude, ∼1 Hz rhythmic activity patterns in both neocortex (nCTX; Steriade et al., 1993; Amzica and Steriade, 1997) and hippocampus (HPC; Isomura et al., 2006; Wolansky et al., 2006). The coordinated slow activity apparent during NREM sleep specifically has been shown to benefit HPC-dependent memory consolidation (Steriade and Timofeev, 2003; Mölle et al., 2004; Marshall et al., 2006). This coordination across nCTX and HPC may be a platform for staged synchronization of neuronal ensembles important for a memory trace, suggesting that the SO in particular may at least partially underlie sleep-dependent memory consolidation (Buzsáki, 1996, 1998; Dickson, 2010). Coordinated and repetitive activity may also be an excellent platform for enhancing synaptic efficacy, an important factor in memory formation (Lee and Wilson, 2002; Dan and Poo, 2004; Buzsáki and Watson, 2012).

Despite its eminent importance for memory, how the SO is coordinated across the nCTX and HPC remains a mystery. Recording activity throughout HPC cell laminae revealed that SO power was maximal at stratum lacunosum-moleculare (SLM) for both field (voltage) and current source density (CSD) measures (Isomura et al., 2006; Wolansky et al., 2006). In addition, the SO peak shows a good degree of coherence between nCTX and the HPC at the level of SLM, which is the site of termination of the temporoammonic pathway from layer III of the entorhinal cortex (EC). It has been widely assumed that this entorhinal input is the main source of the phasing, coordination, and perhaps even the generation of the HPC SO (Isomura et al., 2006; Wolansky et al., 2006). However, a second major input to the SLM also exists, distinct from the multisynaptic corticoentorhinal–HPC pathway, that arises from the midline thalamic nucleus reuniens (RE; Herkenham, 1978). The RE constitutes a robust anatomic link between medial prefrontal cortex (mPFC) and HPC (Vertes et al., 2006, 2007), with a population of cells that project via axon collaterals to both structures, providing a direct disynaptic link between these two important episodic memory regions (Hoover and Vertes, 2012; Varela et al., 2014). While the output of the RE is wholly excitatory (Wouterlood et al., 1990), the input at the level of SLM also activates local inhibitory interneurons, such that the RE can yield both feedforward excitation and inhibition to influence HPC population activity (Dolleman-Van der Weel and Witter, 2000).

Considering this strong disynaptic connection between mPFC and HPC, the dialogue between which is critical for episodic forms of memory (Damasio, 1989; Laroche et al., 2000; Jin and Maren, 2015), more recent work has theorized a role for the RE in coordinating corticohippocampal SO activity (Hallock et al., 2016; Dolleman-van der Weel et al., 2017; Ferraris et al., 2018), which is itself important for memory consolidation (Steriade and Timofeev, 2003; Dickson, 2010; Niethard et al., 2018). In this regard, the RE has been implicated more directly in the consolidation of long-term, episodic forms of memory (Loureiro et al., 2012; Pereira de Vasconcelos and Cassel, 2015; Troyner et al., 2018).

It is clear that the neocortical SO is closely related to hippocampal SO during sleep and sleep-like states (Wolansky et al., 2006; Sharma et al., 2010). What is not clear, however, is how this correspondence is mediated. Here, using state-of-the-art multisite recording techniques, together with optogenetic and chemogenetic manipulations in an *in vivo* rat preparation, we demonstrate that the RE is critically involved in coordinating SO activity between nCTX and HPC. This has marked implications for slow-wave sleep-dependent episodic memory consolidation.

## Materials and Methods

### Animals

Experiments were conducted on 33 male Sprague Dawley rats obtained from the Sciences Animal Support Services and/or Health Sciences Laboratory Animal Services of the University of Alberta with a mean (±SEM) final weight of 426.85 ± 14.41 g. Of these, 7 were used for single- and multiunit recordings; 11 were used for RE and cingulum bundle (CB) stimulation; and 15 were used for chemogenetic inhibition of RE. All animals were provided with food and water *ad libitum* and were maintained on a 12 h light/dark cycle, with lights on at 7:00 A.M. All procedures conformed to the guidelines of the Canadian Council on Animal Care and were approved by the Biological Sciences and/or Health Sciences Animal Policy and Welfare Committees (AUP 092 and AUP 461) of the University of Alberta.

### Materials and Methods

#### Electrodes

Bipolar recording electrodes with tip length separation between 0.5 and 1.3 mm were constructed using Teflon-coated stainless steel wire (bare diameter, 125 μm; A-M Systems). Electrodes were implanted using predetermined coordinates from a stereotaxic atlas, using bregma as a landmark (Paxinos and Watson, 1998). Electrodes were cemented in place using dental acrylic and jeweller’s screws fastened into the skull.

For spatial profile field potential recordings in the HPC, we used a linear 16-contact (100 μm separation) microprobe arranged in a vertical linear array (U-probe, Plexon). The final depth of the probe was determined using the well established electrophysiological profile of theta field activity (Bland and Bland, 1986; Buzsáki, 2002). The position of the multiprobe was histologically confirmed in every experiment by analyzing its track in relation to recorded field activity.

#### Viral vectors

One primary viral vector was used for optogenetic experiments, an adeno-associated virus (AAV; serotype 2/2), expressing a channelrhodopsin-2 variant (ChR2/H134R). It was conjugated with enhanced yellow fluorescent protein (EYFP) and driven by the synapsin promoter (hSyn-ChR2-EYFP). The virus was produced, characterized, and titrated at the University of North Carolina Virus Vector Core Facility (Chapel Hill, NC; ChR2: 3.9 × 10^12^ molecules ml^−1^).

Chemogenetic experiments also used an AAV vector (serotype 2/5) that was also driven by the same synapsin promoter. However, the vector expressed a G_i_-coupled DREADD (designer receptor exclusively activated by designer drug; hM4Di) and was conjugated with both the mCitrine fluorescent protein and a human influenza hemagglutinin (HA) tag (hSyn-hM4Di-HA-mCitrine; 3.5 × 10^12^ molecules ml^−1;^ UNC Virus Vector Core Facility).

Additionally, control experiments were conducted by using a virus with the same promoter (hSyn) and AAV serotype (5) that was coupled only to a fluorescent vector, without any opsin or DREADD (hSyn-mCherry; UNC Virus Vector Core Facility).

#### Photostimulation

An optic fiber (tip diameter, 200 µm) connected to a 473 nm laser (Laserglow Technologies) and calibrated to deliver light at 10–12 mW was positioned to deliver light at intracranial locations. Photostimulation events were driven by a pulse stimulator (Model 2100, A-M Systems) connected to the laser power supply as well as to the analog-to-digital board and PC acquiring data to mark each event (see below).

### Procedures

#### Viral injections and recovery

Rats were initially anaesthetized in a sealed chamber with gaseous isoflurane (4% induction, 1.5% maintenance, in 100% O_2_). After loss of righting reflexes, rats were given an intraperitoneal injection of a ketamine/xylazine cocktail (90 and 10 mg/kg, respectively; Bimeda-MTC, Animal Health; and Rompun, Bayer). Supplemental doses (10% of original) of the ketamine/xylazine cocktail were administered as required to maintain a surgical anesthetic plane. Body temperature was maintained at 37°C following anesthesia using a homeothermic monitoring system (Harvard Apparatus).

Rats were placed into a stereotaxic apparatus (Model 900, David Kopf Instruments) and, using aseptic techniques, were prepared for intracranial injections. A single incision was made along the midline of the scalp, and the skin flaps were pinned back. The skull was leveled by adjusting lambda and bregma to be in the same horizontal plane. Holes were drilled in the skull at predetermined coordinates from a stereotaxic rat atlas (Paxinos and Watson, 1998). Micropipettes (tip diameter, 30 µm) loaded with hSyn-ChR2-EYFP (optogenetic experiments), hSyn-hM4Di-HA-mCitrine (chemogenetic experiments), or hSyn-mCherry (control experiments) were attached to a holder (EHW-2MS, A-M Systems) and lowered using a micropositioner into the brain. Injections targeted either the midline of the nucleus reuniens thalami [anteroposterior (AP), −2.0; mediolateral (ML), +1.9 mm] at an angle 16° oblique to the vertical line to avoid the midline sinus and advanced 6.4–7.2 mm from the brain surface (infusion volume, 400–500 nl) or the infralimbic medial prefrontal cortex [AP, +2.8 to +3.2 mm; ML, +0.7 to +1.1 mm; dorsoventral (DV), −4.4 to −5.8 mm; infusion volume, 300 nl].

Injections were made using a microinjector (PMI-100, Dagan) connected via tubing (PVC, 2.79 × 4.5 mm; Gilson) to the holder, using a pressure of 40 psi and 15 ms pulse length, at a rate of ∼100 nl/min. Micropipettes were left in place for 5–10 min following the injection to allow for adequate diffusion of the virus and to prevent unintended backflow of the viral vector up the pipette track.

Following injection procedures, the scalp was then sutured, and rats were given 0.5 ml of the local anesthetic Marcaine (5 mg/ml, s.c.) around the incision site. Animals were provided with pain medication (meloxicam, 1–2 mg/kg in oral suspension, Boehringer Ingelheim Vetmedica) over a 24 h period postsurgery. Food and water were provided *ad libitum*, and animals were allowed to recover for 2–4 weeks before acute experimentation (see below). Neither the viral injection nor the surgical procedures produced any observable long-term issues.

#### Acute urethane-anesthetized recordings

Rats were initially anaesthetized in a gas chamber with isoflurane in medical oxygen (4% induction, 1.5% maintenance). A catheter was inserted into the femoral vein, and isoflurane was discontinued. General anesthesia was obtained by slow (∼0.03–0.08 ml/min) incremental administrations of urethane (0.4 g/ml) via the catheter. Urethane was chosen because it promotes an unconscious state that closely mimics the typical activity dynamics present during natural sleep, both in terms of brain state alternations as well as in terms of physiologic correlates (Clement et al., 2008; Pagliardini et al., 2013).

Rats were placed into a stereotaxic apparatus. The cranium was exposed by making a single long incision along the scalp and pinning back the skin flaps. The skull was leveled by adjusting lambda and bregma to be in the same horizontal plane. Body temperature was maintained at 37°C using a homeothermic monitoring system (Harvard Apparatus).

##### Unit recording procedures

Bipolar electrodes for recording local field potentials (LFPs) were positioned in the mPFC [AP, +3.2 mm; ML, 0.7 mm; DV (tip of long electrode), −1.2 to −1.8 mm], and straddling the pyramidal layer of CA1 of the dorsal HPC (AP, −3.5 mm; ML, −2.4 mm; DV, −2.5 to −3.5 mm). Our primary index for brain state was determined by the neocortical electrode, but dorsal HPC activity was recorded as an additional confirmation of state. A fine glass micropipette filled with either 1.0 m sodium chloride or 2.0 m sodium acetate, mixed with 2% pontamine sky blue for recording unit activity (resistance ranging from 2 to 10 MΩ) targeted the RE (AP, −2.0 mm; ML, +1.9 mm) at an angle 16° oblique to the vertical line. Micropipettes were mounted on a single-axis fine hydraulic micromanipulator (Model 2660, David Kopf Instruments) that was positioned over the brain with a coarse three-axis manual manipulator (Märzhäuser) and were advanced at a variable rate, as follows: 10–20 μm/s for the first 5 mm; 2–5 μm/s for 5–6 mm; and finally at a rate of 1 μm/s until a final depth typically between 7.5 and 8 mm. The intrapipette solution was in contact with a silver chloride electrode connected to our amplification system.

Local field potentials were amplified in bipolar differential mode at a gain of 1000 and filtered between 0.1 and 500 Hz using a differential AC amplifier (Model 1700, A-M Systems). Single- and multiunit signals were initially amplified at a gain of 10 using a DC amplifier (Neuro Data IR283A, Cygnus Technology). This signal was further amplified using an AC amplifier (Model 1700, A-M Systems) at a gain of 1000 and bandpass filtered between 0.3 and 20 kHz. All signals were digitized and sampled at 20 kHz using a Digidata 1322A analog-to-digital board (Molecular Devices) connected to a PC running the Axoscope acquisition program (Molecular Devices). Unit activity was recorded along the dorsal–ventral axis throughout the RE. At the end of recording sessions, pontamine sky blue was iontophoresed for 5–10 min (+0.4 µA; 7 s on, 7 s off) or pressure injected using a microsyringe attached by tubing to the back end of the pipette.

##### Nucleus reuniens stimulation procedures

Bipolar recording electrodes were positioned in either the anterior frontal cortex (FC) or mPFC (FC: AP, +2.5 mm; ML, −1.2 mm; DV, −0.7 to −1.4 mm; mPFC: AP, +3.2 mm; ML, −0.7 mm; DV, −1.2 to −1.8 mm), as well as in the ipsilateral HPC (AP, −5.5 mm; ML, −4.5 mm). The linear multiprobe was positioned in the contralateral HPC (AP −5.5 mm; ML, +4.5 mm; DV, −3.3 to 4.5 mm) to straddle the CA1 pyramidal layer. Importantly, for monitoring the effects of RE and mPFC stimulation and/or inhibition, the intermediate HPC was consistently targeted using the linear probe (rather than the dorsal HPC in unit experiments; see above), given the prominent projection patterns from RE to this septotemporal region of the HPC (Hoover and Vertes, 2012). Similar to the local field potential recording procedures described above, bipolar recordings were amplified in differential mode at a gain of 1000 and filtered between 0.1 and 500 Hz using an AC-coupled amplifier (Model 1700, A-M Systems). Multiprobe signals were referenced to ground, filtered between 0.1 and 500 Hz, and amplified at a final gain of 1000 via a 16-channel headstage (unity gain) and amplifier (X1000, Plexon). Signals were digitized at a sampling frequency of 1000 Hz with antialias filtering at 500 Hz using a Digidata 1440A Analog-to-Digital Board (Molecular Devices) connected to a PC running Axoscope (Molecular Devices).

On the right side, an optic fiber attached to a 473 nm laser was positioned first above the RE (AP, −2.0 mm; ML, +1.9 mm; DV, −6.4 mm) at an angle 16° oblique to the vertical line. Following stimulation (described below), the optic fiber was removed and repositioned to target the CB (AP, −2.5 mm; ML, +2.7 mm), angled at 40° oblique to the vertical line and advanced 2.6–3.4 mm from the brain surface.

Evoked potentials were produced using 10 ms laser pulses delivered at 1.25 Hz to the RE and then subsequently to the CB, and were averaged over 64 trials. Stimulation trains were delivered during equivalent brain states, specifically during clear deactivated periods characterized by ongoing high power in the 1 Hz signal. This was ensured (1) by noting the ongoing brain state when delivering stimulation trains, and (2) afterward by concatenating all the individual sweeps together, and both visually and spectrally analyzing the underlying baseline activity as a definitive measure of the ongoing brain state.

##### Medial prefrontal cortex stimulation

In a subset of animals (*n* = 8), we used a paired-pulse stimulation paradigm in mPFC sites, with the goal of evoking HPC potentials. The surgical preparation was identical to that used for RE stimulation (see above), except that, instead of targeting an optic fiber over RE or CB, a bipolar stainless steel (0.08 inch bare; 0.11 inch Teflon coated) stimulating electrode was lowered into IL (infralimbic prefrontal cortex) (AP, +2.8 to +3.2 mm; ML, +0.7 to +1.1 mm; DV, −4.4 to −5.8 mm). Following the mPFC stimulation paradigm used by Gemmell and O'Mara (2000), 50–150 μA biphasic current pulses 0.5 ms in duration were delivered with a 30 ms interstimulus interval, every 8 s using a constant current stimulator (Model 2100, A-M Systems). Stimulation epochs were averaged over 32 trials and were always delivered during equivalent brain states.

##### Nucleus reuniens chemogenetic inactivation

In all experiments with mPFC stimulation, rats had been pretreated to express either hSyn-hM4Di-HA-mCitrine or hSyn-mCherry in the RE via our injection procedures above. Subsequent to evoked potential analysis, and after a suitable period of spontaneous recordings, the DREADD agonist clozapine *N*-oxide (CNO; Cayman Chemical) was administered intraperitoneally at a dose of 3 mg/kg (MacLaren et al., 2016). Activity was then recorded for ∼2 more hours, followed by the same mPFC stimulation paradigm described above. This allowed for characterization of the HPC response evoked by mPFC stimulation before and after RE inactivation. We could also then compare baseline activity/coordination between the HPC and mPFC with an intact versus inactive RE within the same recording period.

### Perfusion and histology

Following experimental recordings, 5 s DC pulses of 1 mA using an isolated current pulse generator (Model 2100, A-M Systems) were passed through bipolar recording and stimulating electrodes to generate small electrolytic lesions at their tips. These lesions allowed for subsequent verification of recording and stimulation sites. Rats were then transcardially perfused with 0.9% saline and 4% paraformaldehyde in saline (Thermo Fisher Scientific). The brain was then removed and placed into a 4% formalin and 20% sucrose solution for at least 48 h. Brains were flash frozen using compressed CO_2_ and sectioned with a rotary microtome (1320 Microtome, Leica) at a width of 60 μm. Tissue was countersectioned, with one-third of sections being mounted on gelatin-coated microscope slides for subsequent Thionin staining; another third being mounted on slides and immediately covered using a fluorescence preserving reagent and mounting medium (FluorSave, EMD Millipore); and a third of the tissue saved for immunoreaction for detection of specific neuronal markers.

Immunohistochemistry was performed according to the following protocol. Free-floating sections were rinsed three times using PBS and incubated with 10% normal donkey serum (NDS) and 0.3% Triton X-100 for 60 min to reduce nonspecific staining and increase antibody penetration. Sections were left to incubate overnight with primary antibodies diluted in PBS containing 1% NDS and 0.3% Triton X-100. The following primary antibodies were used: green fluorescent protein (dilution, 1:1000; raised in chicken; Aves Labs); red fluorescent protein (mCherry; dilution, 1:800; raised in rabbit; Millipore); human influenza HA (1:800; raised in rabbit; Cell Signaling Technology); and neuronal-specific nuclear protein (1:800; raised in mouse; Millipore). The following day, tissue was again washed three times with PBS, incubated with secondary antibodies conjugated to the specific fluorescent proteins in each viral construct (Cy2-conjugated donkey anti-chicken; Cy3-conjugated anti-rabbit; Cy5-conjugated anti-mouse; 1:200; Jackson ImmunoResearch), and diluted in PBS and 1% NDS for 2 h. Sections were again washed three times with PBS, mounted, and coverslipped with Fluorsave (EMD Millipore). Microscopic inspection of tissue was used to verify electrode recording loci, optic fiber tracks, and the expression of viral constructs using a Leica DM5500B Fluorescent Microscope.

### Data processing and analysis

Signals were first examined visually using AxoScope 10.6 (Molecular Devices) to choose data segments for further analyses. Analyses were computed and visualized using custom-written code in MATLAB (version R2015b, MathWorks), before being processed with CorelDRAW X6 (Corel). Data analyses included the following: zero phase delay digital filtering, evoked potential averaging, power and phase profile and spectral analyses, coherence, current source density, single- and dual-channel spectra, auto- and cross-correlations, spike-triggered averaging (STA), and spike phase preference. Spectral analysis was used to confirm brain state in chosen segments before conducting other analyses.

#### Field recordings

Autopower, cross-power, coherence, and cross-phase spectra were computed and plotted for both individual and pairs of field signals (details further below). Spectra were estimated from a series of 6-s-long, Hanning-windowed samples with 2 s overlap using Welch’s periodogram method. Power spectrograms (Wolansky et al., 2006; Whitten et al., 2009) were computed using a sliding window procedure, allowing discrete spectra to be calculated at specific time points across the entire time segment. Windows were 30 s in duration, and slid across the entire file in 6 s increments. These discrete spectra were then analyzed as described above. Spectral profiles were also created for activity recorded with the linear multiprobe in the same way, except that each multiprobe channel was compared against a fixed (nCTX or HPC) bipolar reference, and then power values at spectral peak frequency values for both SO and theta states were extracted. The spatial locations of the channels were then estimated based on the power profile for theta (Wolansky et al., 2006), with the phase reversal point being at the interface between stratum pyramidale and stratum radiatum, and the theta maximum being at stratum lacunosum moleculare.

For chemogenetic experiments, 3 min samples of SO activity were extracted based on the analysis of cortical power spectrograms pre- and post-CNO. This duration was selected as a balance between a long-term, stable SO sample, without being compromised by potential nonstationarities. SO time points were all chosen at least 30 min post-CNO injection to ensure adequate time for the ligand or its metabolites to enter the brain (Whissell et al., 2016). A profile of the power at the peak SO frequency for each sample was then created for each channel across the linear multiprobe in the HPC. Using this, we could determine the channel of maximal SO power, which has been previously demonstrated to be the relative location of the SLM (Wolansky et al., 2006). The CSD (described below) profile of the linear multiprobe was then computed. Spectral (particularly power and coherence) estimates were computed as described above, comparing the CSD of the SLM channel to a fixed nCTX or HPC electrode.

##### Single-unit activity

The relationship between neocortical and hippocampal field with RE spikes was assessed by STA. The preferred phase of the unit to the field was computed separately by filtering the field within a specific bandwidth (0.5–1.5 Hz for SO; and 3–5 Hz for HPC theta), and then computing the time points of negative to positive zero crossings. Unit activity was binned (bin size, 18°) according to the phase of the field cycle, from 0° to 360° (from one zero crossing to the next). Spike rates, interspike intervals, and autocorrelation histograms (10–100 ms bin size) were computed to analyze spike train dynamics.

STA significance was computed by comparison with the distribution of STAs using a series (*n* = 100–1000) of randomized (shuffled) spike trains derived from the original data. Spike trains were shuffled using random assignment based on the actual interspike intervals computed for the original spike train. The resulting STA distribution had a variance that was proportional to the amplitude of the original field signal, but that was lower than the original fluctuations for signals with a strong correspondence.

Significance for autocorrelation histograms was computed in a similar way, using the average bin value of individual point processes (single-unit activity) and their fluctuations within a randomized distribution (*n* = 100–1000). The 99% confidence limits were computed as the average value ± 2.6 SEM. We classified units with systematic and periodic fluctuations beyond this window as being rhythmic. Rayleigh statistics for circular data were used to statistically evaluate phase histograms (Zar, 1999).

##### Multiunit activity

Root mean square (rms) envelopes were created for multiunit RE activity by using a 200 ms window slid by 50 ms increments. The resulting envelope was inverted by multiplying by −1 to ensure that it was not antiphase to the ongoing SO. Power, coherence, and phase estimates were computed as described above for field signals. The peak cross-power and coherence frequency was used to estimate the spectral phase angle between the RE rms and field signal. Coherence between the signals was used as a measure of phase preference (radius). To determine a 99% confidence limit for coherence estimates, a series of time-reversed coherence spectra were computed, and the distribution of values across the entire spectrum was assessed.

##### Current source density analysis

CSD analysis was conducted on spontaneously collected field samples or evoked potential averages recorded using the linear multiprobe, following the assumptions of Freeman (1975), Rodriguez and Haberly (1989), and Ketchum and Haberly (1993). Briefly, CSD was computed by estimating the second spatial derivative of adjacent multiprobe voltage traces.

## Results

### Nucleus reuniens neurons phase lock with the slow oscillation during deactivated states

**Figure 1.**
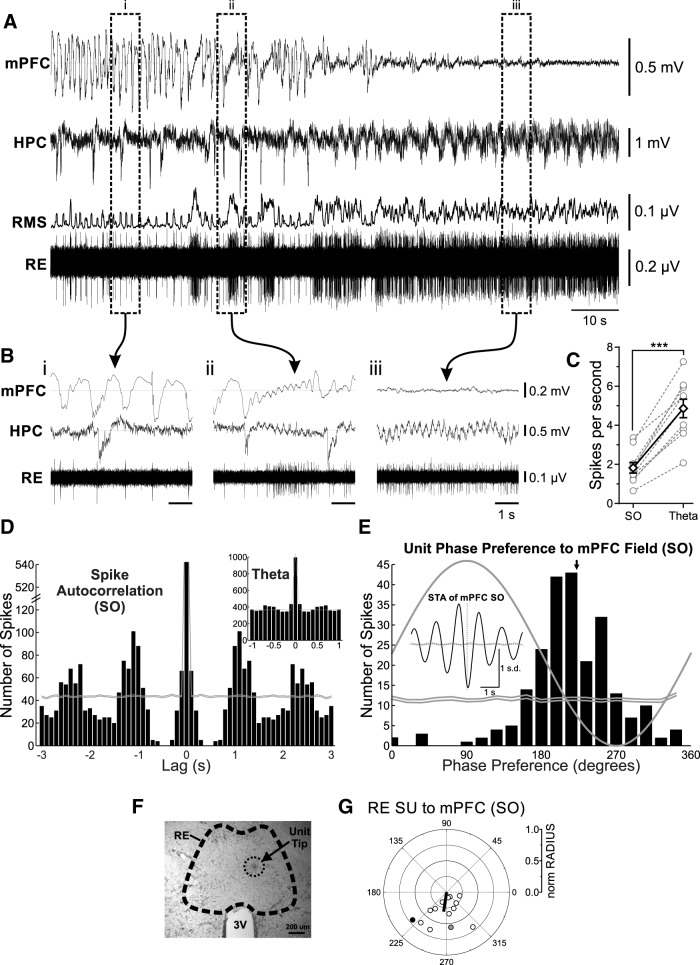
RE units are phase coupled to the forebrain SO. ***A***, Simultaneous field recordings in mPFC and HPC, with glass micropipette single-unit recording in RE, and rms envelope (200 ms window, slid by 10 ms) of RE single-unit recording, during a spontaneous deactivated-to-activated field state change. ***B***, Bracketed segments correspond to expansions, which highlight the features of field and RE unit activity across states. ***Bi***, Deactivated (SO) state showing a slow, rhythmic firing of a representative RE unit, coupled to the negative phase of the mPFC SO. ***Bii***, Transitionary state illustrating the tonic firing of an RE unit only during prolonged activated forebrain activity. ***Biii***, Activated (theta) state illustrating tonic firing of the RE unit. ***C***, Spike frequency measures for RE neurons that were recorded across both deactivated and activated states, showing a prominent increase in firing rate during theta states for individual units (gray hollow circles and dashed gray lines), and on average (black hollow diamonds and solid black lines; Error bars represent SEM. ***p < 0.001). ***D***, Spike train autocorrelation histograms of a representative RE unit during SO and theta (inset), demonstrating significant state-dependent rhythmicity (bin size, 100 ms). Gray lines represent 99% confidence intervals from randomized distributions. ***E***, Phase histogram for this unit during SO, showing a strong preference for the descending/negative phase of the mPFC SO (bin size, 18°). Black arrow indicates mean phase preference. Inset, Spike-triggered average of the RE unit firing to mPFC field during SO. ***F***, Photomicrograph of a representative coronal tissue section, showing the expression of pontamine sky blue localized to the RE, indicating the tip of the single-unit recording pipette. ***G***, Circle plot of the preferred phase of RE units to mPFC SO cycle. The black line indicates the mean angle (°) and strength of phase preference (Rayleigh statistics). Gray, filled circle corresponds to the unit shown in ***A*** and ***B***. Black, filled circle corresponds to the unit shown in ***D*** and ***E***. SU, Single unit.

Striking changes in spike train dynamics of verified RE single unit recordings (*n* = 7 rats) were observed across activated (theta) and deactivated (SO) states (Fig. 1). Every RE neuron sampled across both states (10 of 10) changed its pattern of activity concomitantly with forebrain state (Fig. 1*A*, see example). During activated states, neuronal discharge was consistently elevated (average frequency, 4.86 ± 0.47 spikes/s; Fig. 1*Biii*) and was significantly higher than that observed during SO states (1.83 ± 0.27 spikes/s; *p* < 0.0001, two-tailed paired *t* test; Fig. 1*Bi*,*C*). In addition to firing frequency, the pattern of discharge appeared to be tonic during theta and rhythmic during SO. Using autocorrelation analysis, we observed that in all cells recorded during the SO state (*n* = 14), there was clear rhythmicity (average, 0.86 ± 0.06 Hz), which was highly similar (*p* = 0.97, two-tailed paired *t* test) to the average frequency (0.86 ± 0.05 Hz) of the cortical SO. No such rhythmicity was apparent in the randomized (spike-shifted) distribution. In the 10 cells recorded across both states, no rhythmicity was observed during the theta state (Fig. 1*D*, inset). Indeed, and as shown in Figure 1*D*, the randomized (spike-shifted) distribution was highly similar to the original histogram.

To determine whether there was any relationship between RE unit activity and the ongoing SO, we performed a cycle-by-cycle field potential phase analysis of spiking behavior as well as spike-triggered averaging of the local field potential at both mPFC and HPC sites. Using the phase of the ongoing cortical SO LFP to organize unit activity (Fig. 1*E*), we observed a prominent and highly significant degree of phase coupling of unit discharge to the field potential oscillation (for this example, the average preferred angle, 219°; Rayleigh distribution, *z* = 110.75; *n* = 229, *p* < 0.0001). This relationship was not at all apparent in the randomized (spike-shifted) distribution. Across all RE cells, there was significant (as determined by Rayleigh statistics) coupling in 12 of the 14 total cells, and the average preferred angle was also consistent across cells at a phase just before the negative peak of the SO (overall average angle, 261.59°; overall average radius, 0.31; *F*_(2,12)_ = 17.61, *p* < 0.001; Fig. 1*G*). A similar degree of coupling was observed to the ongoing HPC SO, although only eight cells showed a significant phase preference (overall average angle, 14.07°; overall average radius, 0.086; *F*_(2,12)_ = 4.17, *p* < 0.05). The HPC analysis was complicated by the fact that the bipolar montage was optimized for theta, and not for local SO, which minimized this latter signal in many of our experiments (also shown by lower than usual coherence analysis with the neocortical SO).

We confirmed the same phasic relationship using spike-triggered averaging (Fig. 1*E*, inset). The field potential average, organized by RE unit spiking, was highly rhythmic and showed a prominent negativity time locked to RE unit discharge. This relationship was flat when the averaging was done with randomized spike-shifted timing (Fig. 1*E*, inset). In 12 of the 14 recorded units, a similar phasic relationship to the HPC SO was found. In the remaining two examples, the hippocampal LFP site was not optimized for SO (as above).

**Figure 2.**
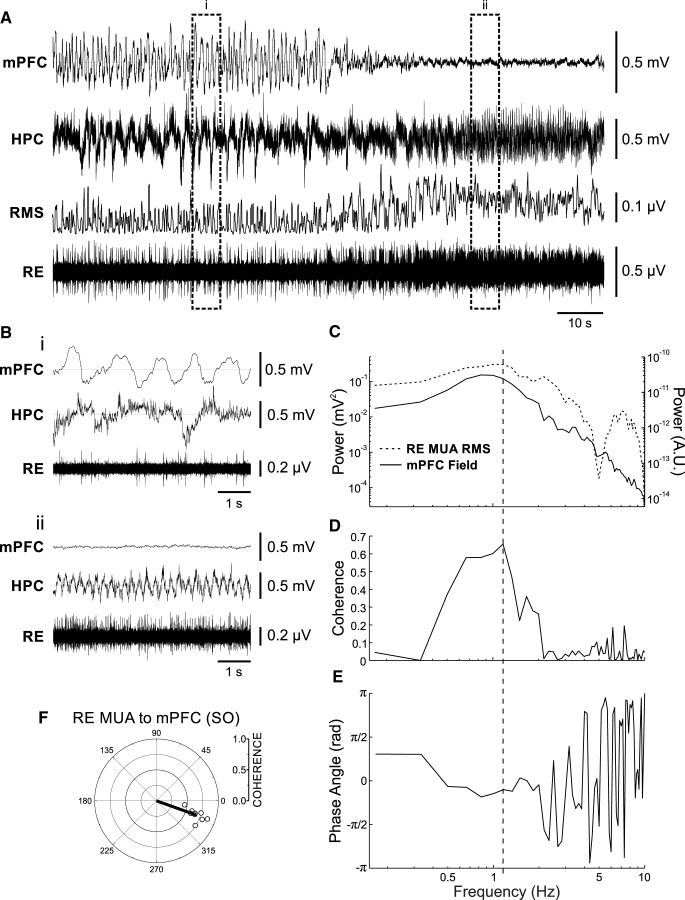
RE MUA is phase coupled to the forebrain SO. ***A***, Simultaneous field recordings in mPFC and HPC, with glass micropipette multiunit recording in RE, with rms envelope (200 ms window, slid by 50 ms) of RE multiunit recording above, during a spontaneous deactivated-to-activated field state change. Bracketed segments correspond to expansions in ***B***, which highlight the features of field and RE unit activity across states. ***Bi***, Deactivated (SO) state showing a slow, rhythmic firing of representative RE multiunit activity, coupled to the negative phase of the mPFC SO. ***Bii***, Activated (theta) state illustrating tonic firing of RE units. ***C***, Autospectral power of mPFC field (black line, left axis) and of the rms envelope of RE MUA (dashed black line, right axis) during SO. ***D***, ***E***, Coherence (***D***) and phase spectra (***E***) across the two signals shown in ***C***. Vertical dashed line through ***C–E*** indicates SO frequency extracted from spectra. ***F***, Circle plot of preferred phase as measured by coherence (radia) and phase angles (°) obtained from spectra of RE multiunit activity compared to the mPFC SO cycle. Shaded gray circle corresponds to the multiunit activity illustrated in all panels of this figure.

In another nine recording situations, we were able to record multiunit activity (MUA) from the RE to gather a population index of unit activity (Fig. 2). Similar to the single-unit data, MUA tended to show tonic, high-frequency patterns during theta (Fig. 2*A*,*Bii*), and slow, rhythmic bursts during SO (Fig. 2*A*,*Bi*). By using the rms envelope of this activity (Fig. 2*A*; see Materials and Methods), we were able to compare it to the ongoing forebrain SO using spectral analysis. In nine of nine cases, and as shown in Figure 2*C–E*, we determined that the MUA had a significant phase relationship to ongoing mPFC SO by coherence analysis at the frequency peak demonstrated in the cross-power spectrum (values >0.18 were determined to be significant via time-shifted randomizations; see Materials and Methods). By examining the cross-phase spectrum at this coherent frequency, we could determine the average preferred phase of the peak positivity of the MUA rms to the ongoing phase of the mPFC SO (Fig. 2*E*). On average, and as shown in Figure 2*F*, this occurred just after the peak negativity in the cortical field oscillation (average angle, 340.34°; radius, 0.68; *F*_(2,7)_ = 141.11, *p* < 0.001; Fig. 1*H*). There was also a slight phase preference of the peak positivity of the RE MUA rms to the negative trough of HPC SO activity (average angle, 248.35°; radius, 0.12).

Our unit recordings showed that the RE does exhibit SO-related activity that was correlated to ongoing forebrain SO, and could thus carry this signal between cortex and hippocampus.

### Optogenetically stimulating the nucleus reuniens generates an evoked potential with maximal current sink in the stratum lacunosum moleculare of hippocampus

**Figure 3.**
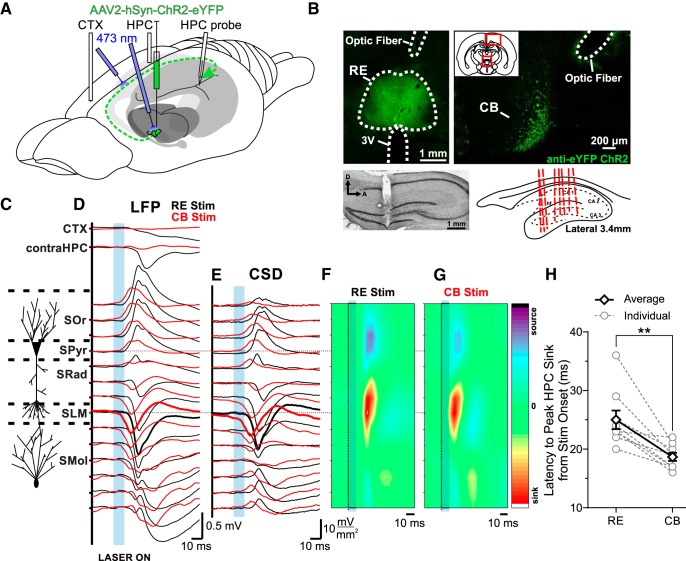
Optogenetic stimulation of the RE/CB reliably produces an evoked potential in the HPC. ***A***, Schematic illustration of the injection, stimulation, and recording procedures. Schema modified from Amaral and Witter (1995). ***B***, Top left, Representative coronal tissue section showing the expression of hSyn-ChR2-EYFP virus localized to RE, with optic fiber track positioned dorsal to RE. Top right, hSyn-ChR2-EYFP viral expression in the CB from a single injection into RE, with optic fiber track. Inset, Schematic of relative locations of top two images. Bottom left, Nissl-stained sagittal section of HPC illustrating multiprobe track penetrating cell lamina perpendicularly. Bottom right, Schematic sagittal illustration of all (except one) multiprobe tracks through the HPC. ***C***, Schematic depiction of HPC cell lamina as determined by theta profiles. ***D***, Local field potentials evoked in frontal CTX, contralateral HPC, and in the ipsilateral HPC via the multiprobe. Laser light delivery (10 ms pulse at 473 nm wavelength; blue rectangle) to the RE (black lines) produced a response in CTX and contralateral HPC, as well as a profile that reversed from dorsal-to-ventral locations through the ipsilateral CA1 region of the HPC, with a maximal amplitude at a depth corresponding to SLM (which was also the power maximum of the SO and theta profiles). Identical stimulation of the CB (red lines) produced a similar but lower latency profile in ipsilateral HPC, but largely eliminated the evoked response in CTX and contralateral HPC, indicative of the specificity of the ipsilateral RE pathway through the CB. ***E***, Current sink/source density line plots illustrating net synaptic current flow at each ipsilateral hippocampal recording site, showing a maximal sink at SLM following both RE (black) and CB (red) stimulation (sinks are shown by downward or negative deflections, sources by upward or positive deflections). ***F***, ***G***, Color contour plot of the CSD traces in ***E*** following RE (***F***) and CB (***G***) stimulation (marked by a dashed line and blue box), depicting a large sink centered on SLM, with a corresponding source in basilar regions of CA1. CSD scales are −17 to 17 mV/mm^2^ for RE stimulation, and −11 to 11 mV/mm^2^ for CB stimulation. ***H***, Latency from the end of the 10 ms optical stimulation to peak HPC sink, which in every case was at SLM, for individual animals (gray hollow circles and dashed gray lines), and on average (black hollow diamonds and solid black lines). Error bars represent SEM. (**p < 0.01). SOr, Stratum oriens; SPyr, stratum pyramidale; SRad, stratum radiatum; SMol, stratum moleculare.

Having determined that RE neurons themselves displayed prominent SO activity and were coupled to ongoing SO, we next wanted to assess the influence of stimulating RE neurons on HPC activity. To do this, we adopted an optogenetic approach that involved targeted stereotaxic viral delivery to the RE and then subsequent simultaneous depth potential recording in the HPC during light stimulation directed at either the RE or its specific unilateral pathway to the HPC via the cingulum bundle (Fig. 3).

Following hSyn-ChR2-EYFP viral infection in the RE in nine rats, a series of optical pulses was delivered (see Materials and Methods) directly to the RE, during 14-channel linear probe recording through the CA1 region of HPC together with bipolar LFP recordings from the contralateral HPC and frontal cortices (Fig. 3*A*). Viral infection and electrode placements were histologically confirmed following every experiment (Fig. 3*B*). In all cases reported, viral infection was largely confined to the RE, with occasional minimal expression in the dorsally adjacent rhomboid nucleus. This, in part, motivated our subsequent stimulation of the CB, which confirmed that the evoked response in HPC was truly RE mediated. All electrode tracks were localized to CA1, extending through stratum pyramidale and SLM and into the molecular layer of the dentate gyrus. The resulting evoked potential in the HPC was averaged over 64 individual stimulations and revealed a large negative potential that was maximal at a depth corresponding to the SLM (Fig. 3*C*,*D*). CSD analysis revealed a prominent sink (−7.89 ± 2.44 mV/mm^2^) at an average latency of 25.00 ± 1.60 ms from the onset of stimulation at the level of the SLM (Fig. 3*E*,*F*). This potential profile is consistent with previous reports of RE-mediated potentials in the HPC (Dolleman-Van der Weel et al., 1997) and is also consistent with the projections of the RE to the HPC, which synapse on the distal dendrites of CA1 pyramidal cells (Herkenham, 1978).

To isolate this input more specifically, we directed optical stimuli at the level of the ipsilateral CB, the pathway by which the RE projects to the HPC (Herkenham, 1978; Wouterlood et al., 1990). The evoked LFP profile during CB stimulation closely resembled the direct RE stimulation, again showing a maximal negativity at SLM (Fig. 3*D*). This evoked potential was limited to the ipsilateral hippocampus, and any influence of RE stimulation on contralateral HPC and neocortical potentials was markedly reduced or completely eliminated (contralateral HPC amplitude decreased 90.8%, *p* = 0.0035; neocortical amplitude decreased 93.0%, *p* = 0.0051; two-tailed paired *t* tests) using this procedure. Subsequent CSD analysis revealed a sink in the SLM similar to that evoked by RE stimulation, albeit at a significantly reduced latency of 18.67 ± 0.69 ms (*p* = 0.0020, compared with RE stimulation, two-tailed paired *t* test) and magnitude (−4.51 ± 1.36 mV/mm^2^; *p* = 0.024, two-tailed paired *t* test with respect to RE stimulation; Fig. 3*E*,*G*,*H*).

### Stimulating the medial prefrontal cortex generates an evoked potential in the hippocampus that is mediated via the nucleus reuniens

**Figure 4.**
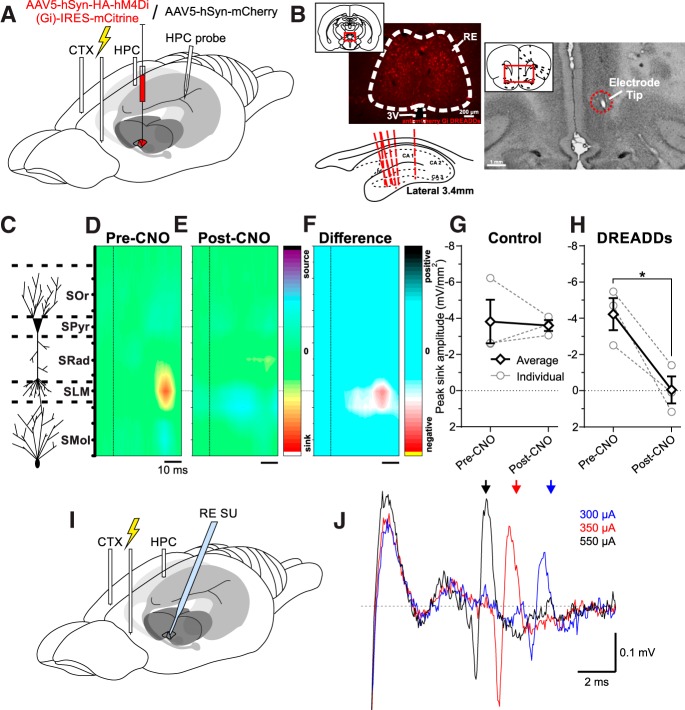
Stimulation of the mPFC produces an evoked response in the HPC that can be blocked by selective RE inhibition. ***A***, Schematic illustration of the injection, stimulation, inhibition, and recording procedures. ***B***, Top left, Representative coronal tissue section showing expression of hSyn- hM4Di-HA-mCitrine virus localized to RE. Scale bar, 200 μm. Inset, Schematic of relative location of image. Top right, Representative coronal Nissl-stained tissue section showing the tip of a stimulating electrode in IL. Inset, Schematic of relative location of image. Bottom left, Schematic sagittal illustration of all (except one) multiprobe track through HPC. ***C***, Schematic depiction of HPC cell lamina. ***D***, Color contour plot of ipsilateral HPC CSD following the second pulse of paired-pulse stimulation (dashed vertical line) in IL before CNO administration, illustrating a prominent sink localized to SLM. CSD scales are −6 to 6 mV/mm^2^. ***E***, Color contour plot of CSD following identical IL stimulation paradigm (dashed vertical line), post-CNO administration in a DREADDs animal, illustrating a disappearance of the SLM sink. CSD scales are −6 to 6 mV/mm^2^. ***F***, Difference contour plot created by subtracting the post-CNO CSD in ***E*** from the pre-CNO CSD in ***D*** from the same animal, illustrating that the difference between the two conditions is limited and localized to the SLM sink. Difference scales are −7.4 to 7.4 mV/mm^2^. ***G***, Peak sink amplitude pre-CNO and post-CNO in control (hSyn-mCherry) animals (individuals: gray hollow circles and gray dashed lines; average: black hollow diamonds and solid black lines), showing no significant difference in amplitude between conditions. ***H***, Peak sink amplitude pre-CNO and post-CNO in DREADDs (hSyn-hM4Di-HA-mCitrine) animals (individuals: gray hollow circles and gray dashed lines; average: black hollow diamonds and solid black lines), illustrating a robust decrement in sink amplitude after CNO administration in all animals (Error bars represent SEM. *p < 0.05). ***I***, Schematic illustration of the mPFC stimulation and RE single-unit (SU) recording procedures. ***J***, Increasing stimulation intensity (blue trace, 300 μA; red, 350 μA; black, 550 μA) in IL decreases the latency of responding in RE single-unit and multiunit activity, indicating direct mPFC–RE orthodromic excitation. Arrows, Average spike latency over 10 stimulation trials.

Given the dense projections from PL and IL to RE (Vertes, 2002; Mathiasen et al., 2019), we targeted these sites in *n* = 7 rats (verified histologically; Fig. 4*B*) using a paired-pulse stimulation paradigm to record its influence in the HPC (Fig. 4*A*). In *n* = 4 animals, a G_i_-coupled DREADD, hM4Di virus (Armbruster et al., 2007; Atasoy and Sternson, 2018) was preinjected into the RE 3 weeks before experimentation; in the remaining three animals, a control mCherry vector was used. This allowed us to evaluate the influence of chronic silencing of RE neurons following systemic CNO administration (Whissell et al., 2016; Aldrin-Kirk and Bjorklund, 2019). We then evaluated the evoked potential profile pre- and post-CNO administration. As with direct RE stimulation, we limited our analysis to those trials occurring during deactivated brain states.

Stimulating IL yielded an evoked potential profile in the HPC that, following CSD analyses, resembled that evoked by RE and CB stimulation (Fig. 4*C*,*D*). In control (mCherry) animals, a maximal current sink was observed at 17.67 ± 0.88 ms pre-CNO and at 17.67 ± 1.76 s post-CNO. This latency is shorter when compared with the RE or CB stimulations as a consequence of using electrical, rather than optogenetic, stimulation. In a pilot chemogenetic inactivation experiment, the IL was optogenetically stimulated and evoked a response in HPC with a latency of 52 ms. Neither the latency to the peak sink (*p* = 1.00, two-tailed paired *t* test) nor the magnitude (*p* = 0.84, two-tailed paired *t* test; −3.79 ± 1.20 mV/mm^2^ pre-CNO, and −3.57 ± 0.30 mV/mm^2^ post-CNO) across conditions was significantly different (Fig. 4*G*).

In DREADD-expressing animals, the maximal sink amplitude was significantly (*p* = 0.048, two-tailed paired *t* test) decreased following CNO administration, from −4.22 ± 0.89 to −0.034 ± 0.75 mV/mm^2^ (Fig. 4*E*,*H*). Given the robust diminution of the sink post-CNO, the current flow at an equivalent time point to that chosen pre-CNO was selected for comparison. Subtracting the entire evoked CSD potential post-CNO from that pre-CNO revealed an obvious abolition of the effect of prefrontal stimulation on the HPC (Fig. 4*F*).

These results imply that SO-related activity in the mPFC is actively transmitted to the HPC via excitation of RE neurons. To show this directly, we recorded single-unit and multiunit unit activity in RE during mPFC stimulation (*n* = 6). Indeed, we found that mPFC stimulation evoked orthodromic excitation in RE units (Fig. 4*I*,*J*).

### Chemogenetic silencing of the nucleus reuniens impairs prefrontal–hippocampal SO coordination

**Figure 5.**
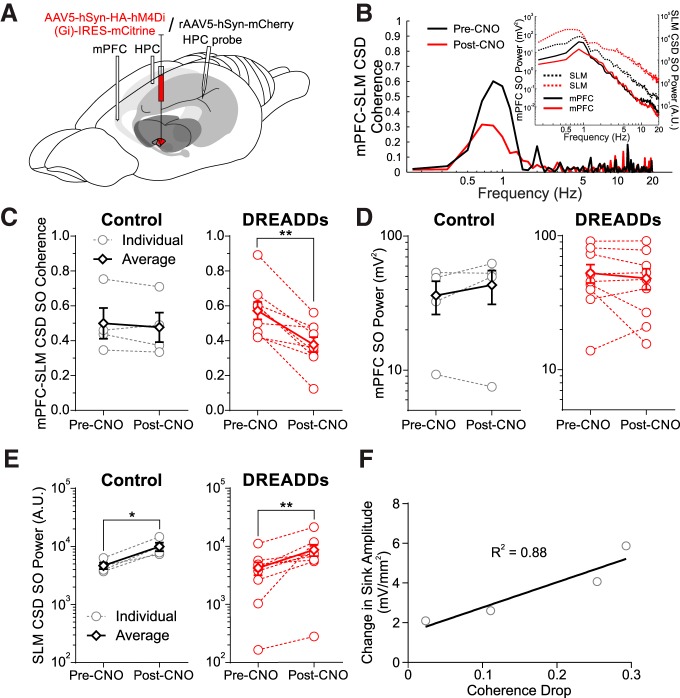
RE inhibition decreases mPFC–HPC SO synchrony. ***A***, Schematic illustration of the injection, inhibition, and recording procedure. Either a hSyn-hM4Di-HA-mCitrine (DREADDs) or an hSyn-mCherry (CONTROL) virus was injected into RE 3 weeks before experimentation. ***B***, Example difference in mPFC-SLM CSD coherence pre-CNO and post-CNO in a representative DREADDs animal. Black lines, pre-CNO; red lines, post-CNO. Inset, Autospectral power of mPFC field (solid lines, left axis) and SLM CSD (dashed lines, right axis). ***C***, Coherence between mPFC bipolar field and HPC CSD (selecting the SLM depth trace in particular) in both control (left: individuals: gray hollow circles and gray dashed lines; average: black hollow diamonds and solid black lines) and DREADDs (right: individuals: red hollow circles and red dashed lines; average: red hollow diamonds and solid red lines) groups, showing a significant drop in every DREADDs animal, but not in controls. ***D***, Autospectral power in mPFC at the peak SO frequency in CONTROL (left) and DREADDs (right) groups, showing no significant difference in either. ***E***, SLM CSD power at the peak SO frequency in CONTROL (left) and DREADDs (right) groups, showing an increase in power across both conditions. ***F***, Scatterplot of the reduction in evoked sinks via mPFC stimulation as a function of the drop of SO coherence following chemogenetic RE inhibition. Solid line indicates least-squares regression fit (*R* ^2^ = 0.88). Error bars represent SEM. *p < 0.05; **p < 0.01.

In the same animals expressing a G_i_-coupled DREADD virus in the RE (*n* = 9) or control vector (mCherry *n* = 4; Fig. 5*A*), we evaluated the coordination of the SO across mPFC and HPC sites pre-CNO and post-CNO administration. To minimize any contamination of the hippocampal signal by volume conduction from overlying cortex, we used the continuous CSD signal at the SO maximal contact on the linear probe (Wolansky et al., 2006). The average coherence of the SO signal during baseline recordings was 0.50 ± 0.09 in controls and 0.57 ± 0.05 in DREADD-infected animals (Fig. 5*C*). This was significantly reduced following CNO administration in every animal in the DREADD-infected group (0.38 ± 0.04; *p* = 0.0011, two-tailed paired *t* test; Fig. 5*B*, see example), while no significant difference was observed in the control group (0.48 ± 0.08; *p* = 0.35, two-tailed paired *t* test; Fig. 5*B*,*C*). As coherence can be influenced by the relative power of the frequency of interest at the two sites (Srinath and Ray, 2014), we ensured that there were no reductions of SO power in either the mPFC or HPC sites. There was no reduction of field SO power in the mPFC across these time points (control, *p* = 0.24; DREADDs, *p* = 0.26; two-tailed paired *t* tests; Fig. 5*B*,*D*). Interestingly, CSD SO power in the HPC increased post-CNO (control, *p* = 0.023; DREADDs, *p* = 0.010; two-tailed paired *t* tests) between pre-RE and post-RE inhibition conditions (Fig. 5*E*). This increase at SO frequencies in the CSD signal from the linear probe was also present in three additional experiments in which no CNO was administered and was likely a nonspecific result related to the passage of time itself, since we also observed a nonspecific increase in broadband (0–500 Hz) power as well.

In four animals, we could relate the drop in SO coherence to the reduction of the evoked sink from mPFC stimulation. The degree of suppression in the evoked potential was positively related to the reduction in coherence, with an *R* ^2^ value of 0.88 (Fig. 5*F*). Thus, any reduction of the RE-mediated mPFC stimulation effects on the HPC was directly related to the subsequent reduction in SO coordination across mPFC and the HPC.

## Discussion

Our data show that the connections mediated between medial prefrontal cortex and the hippocampus via the nucleus reuniens appear to be necessary for coordination of the slow oscillation between these structures. Indeed, we showed that RE neurons track the ongoing slow oscillation and that they have consistent phase relationships to both the neocortical and hippocampal expression of this activity. When RE cells are inactivated, communication between the mPFC and HPC is disrupted, and so too is the coherence of the corticohippocampal SO. Given the role of slow-wave sleep, and in particular the SO, in memory consolidation, we feel that the RE is in a prime position to influence the coordination of cellular ensembles that would be relevant for the solidification of episodic memories.

### Urethane anesthesia and natural sleep

Spontaneous, rhythmic brain state alternations under urethane anesthesia bear more than a passing resemblance to those across REM and NREM sleep. Field potential recordings from neocortical and hippocampal sites demonstrate transitions between an activated pattern composed of low-power, high-frequency cortical activity together with rhythmic hippocampal theta frequency (3–4 Hz) activity versus a deactivated pattern of high-amplitude, slow-frequency (∼1 Hz) activity at both sites. The timing of these alternations overlaps very well with those observed in natural sleep, and concomitant with these state alternations are peripheral physiologic changes, including respiratory rate, heart rate, muscle tone, and temperature. These correspond well to observed changes across natural REM and NREM states (Clement et al., 2008; Whitten et al., 2009; Pagliardini et al., 2012). Urethane-anesthetized mice also show these same alternations (Pagliardini et al., 2013). As we have also reviewed recently (Ward-Flanagan and Dickson, 2019), other central and peripheral changes observed across these state changes also correspond well to those noted for natural sleep. As such, the urethane-anesthetized rodent is a highly tractable model that is widely used to study central and peripheral dynamics of natural sleep.

### Activity states of nucleus reuniens neurons

The activity of RE neurons has been previously examined almost exclusively during theta. Consistent with previous work (Morales et al., 2007; Lara-Vásquez et al., 2016; Walsh et al., 2017), we found that during activated/theta brain states, RE units showed high-frequency tonic activity that had no rhythmicity or oscillatory LFP correlates. During transitions to deactivated states, and especially during strong forebrain SO, however, the activity of all RE neurons recorded developed striking phase rhythmicity, strongly coupled to ongoing SO. Not only does this suggest a strong state-dependent modulation of information processing by the RE but also implicates it in following and potentially transmitting SO rhythmicity from the mPFC to the HPC.

The RE sits in a nodal region interfacing mPFC and HPC. RE neurons receive input from mPFC and, in turn, send a dense excitatory projection to SLM of CA1 (Herkenham, 1978; Yanagihara et al., 1987; Wouterlood et al., 1990). Previous work (Isomura et al., 2006; Wolansky et al., 2006) has demonstrated that HPC SO potentials and current sink–source alternations have the strongest power at the level of the SLM. These results suggest that inputs arriving at the SLM are the source of phasing, coordination and, perhaps, the generation of the SO in the HPC. Although previous work implicated layer III neurons of the entorhinal cortex in mediating this SO activity, via the densely SLM-innervating temporoammonic pathway (Isomura et al., 2006; Wolansky et al., 2006; Sullivan et al., 2011; Hahn et al., 2012; Taxidis et al., 2013), our present data suggest a more important role for the RE. Indeed, the RE may well integrate SO activity at both the level of HPC and EC through its additional prominent connections to EC layer III. This latter idea remains to be tested.

Our unit findings are also consistent with other work conducted during SO activity facilitated by ketamine anesthesia (Angulo-Garcia et al., 2018; Ferraris et al., 2018). This work also showed strong SO coupling of RE units but was focused more specifically on examining ON-phase gamma coupling between the HPC and mPFC that might be mediated by the return projections of the RE. Indeed, the grouping of ensemble activity into the active (ON) phase of the SO would be a powerful way to enhance synaptic coupling between sets of neurons in disparate episodic memory regions, and this may be the exact mechanism by which SO activity enhances episodic memory consolidation. Although Ferraris et al. (2018) suggested that the RE mediated coupling between the HPC and the mPFC (i.e., in the opposite direction to what we propose), it very well may be the case that it does both, and thereby could mediate continuing reverberative activity in this bidirectional circuit. Of course, this would presumably strengthen cellular assemblies to an even greater degree across mPFC and HPC.

The mPFC (and the IL in particular) has also been shown to alter the firing of RE cells (Zimmerman and Grace, 2018). Our findings suggest that the mPFC provides a rhythmic input to RE during SO states, the timing of which is then relayed to CA1. As such, it is likely that the RE is entrained by slow, rhythmic stimulation via the mPFC, which could subsequently entrain the HPC. Indeed, we observed that increased forebrain deactivation predicted increased rhythmicity in the RE, suggesting that SO activity in mPFC is reliably transmitted to, and likely entrains, RE units to the cortical SO ON phase. Moreover, we also showed that mPFC stimulation orthodromically excited RE neurons. In our model, we regard the RE as a rhythmic mediator in a disparate slow-wave circuit, not responsible for generating the SO in either mPFC or HPC per se, but rather for maintaining synchronous coordination between them through an SO-driven clock-like mechanism.

### Disrupting mPFC coupling to the HPC by RE inactivation decouples forebrain so coordination

We confirmed that optogenetic activation of RE inputs to HPC evoked an excitatory sink at the level of the SLM, similar to previous electrical stimulation studies (Dolleman-Van der Weel et al., 1997, 2017; Bertram and Zhang, 1999; Morales et al., 2007). Furthermore, stimulation of mPFC itself could also produce this same pattern of activation in the HPC, which was then abolished by chemogenetic inactivation of RE neurons. In the same inactivation experiments, this reduction of the evoked response was strongly related to a marked decrease in mPFC–HPC SO phase coherence, but without any loss of SO power at either site. Altogether, our experiments strongly suggest that SO coupling (but not power) across mPFC and HPC is mediated by the interfacing activity of RE neurons.

Of interest here is the mediation of remaining slow power at the level of the HPC following RE inactivation. The power profile of SO activity remained similar, suggesting that activity at SLM continues to drive slow oscillatory activity within the hippocampal circuit. With the RE inactive, the only other input would be the temporoammonic pathway from EC. Further work will determine the role of the layer III input from EC and how it integrates with RE activity.

It is possible that RE activation (either directly or via mPFC stimulation) evokes a response in the HPC via a complex interaction with the entorhinal cortex. Anterograde tracing of outputs from the RE showed not only dense labeling in the SLM, but in the medial entorhinal cortex as well (Wouterlood et al., 1990). In this study, transecting the CB showed large numbers of degenerating axon terminals present in medial entorhinal cortex, suggesting that the RE may simultaneously influence the parent cell bodies of the perforant pathway, as well as target neurons in CA1. More recent work has examined the effect of coincidental stimulation of both RE and entorhinal cortex (Dolleman-van der Weel et al., 2017). Stimulating both sites produced a nonlinear interaction throughout the HPC evoked potential, suggesting that RE and EC axons synapse, at least partly, onto the same dendritic sites in CA1 pyramidal cells. The authors posit that the role of the RE in a slow-wave-related circuit may be to facilitate an entorhinal–HPC dialogue, and that is what may be responsible for coordinating and synchronizing the SO between frontal cortical and HPC sites (Dolleman-van der Weel et al., 2017). A natural follow-up to the present study then, is to assess any changes in SO coherence between the entorhinal cortex and HPC following RE inactivation or excitation. What we can conclude from our work is that the RE certainly has an excitatory influence on the HPC, and that without that input, prefrontal–HPC SO synchrony is considerably impaired.

### The RE as a nodal hub between mPFC and HPC for synchrony and memory

Consistent with the idea of the RE acting as a gate or nodal hub for information flow between mPFC and HPC (Vertes et al., 2007; Cassel et al., 2013; Varela et al., 2014), the role of the RE in a host of memory tasks is being increasingly recognized. RE lesions or inactivation have a particularly detrimental effect on spatial working memory tasks requiring interactions between mPFC and HPC, but not those that only require HPC activity alone (Davoodi et al., 2009; Hembrook and Mair, 2011; Hembrook et al., 2012). The RE also has a described role in determining the generalization and specificity of contextual fear memories (Xu and Südhof, 2013). A critical role of the RE in forming long-term (>24 h) memories, but not acquisition or short-term memories, has also been demonstrated (Loureiro et al., 2012; Barker and Warburton, 2018). Importantly, however, many studies have used behavioral tasks that assess influences of RE impairments at only short delays or periods in which animals may not have slept, highlighting that the RE is not solely involved in slow-wave coordination for off-line memory consolidation, but may also be involved in memory during on-line theta states (Hallock et al., 2016; Maisson et al., 2018). Together, these results demonstrate a role for the RE in the integration of HPC- and mPFC-mediated memory function.

More recent considerations of nCTX–HPC dynamics have discussed the role of the RE in mediating widespread coordination (Hallock et al., 2016; Dolleman-van der Weel et al., 2017; Ferraris et al., 2018). Specific studies have intimated that the directionality of information transfer in this tripartite circuit depends on the specific stage of the task involved (Spellman et al., 2015; Hallock et al., 2016). For instance, in a delayed alternation T-maze task HPC theta activity led and organized both unit and theta activity in the mPFC during the delay period between trials (Hallock et al., 2016). Conversely, during choice point traversals, slow gamma activity in the mPFC led and predicted slow gamma activity in the HPC. Effective performance of the task as such requires an interplay between the mPFC relaying choice information to the HPC, which stores and subsequently relays this information back to mPFC to guide future decision-making. Critically, communication in either direction was eliminated following RE inactivation. Interestingly, and in support of our directional model of SO influence from the mPFC to the HPC, another recent study demonstrated that statistical (using partial correlation analysis) or pharmacological removal of the RE impaired PFC–HPC coherence in a 2–5 Hz bandwidth, but had minimal effect on theta coupling, which the authors posit is because of a strong, intact connection directly from HPC to mPFC (Roy et al., 2017). In contrast, Ferraris et al. (2018) describe an HPC-to-mPFC directionality during slow gamma activity that is disrupted by muscimol infusion into the RE. Interestingly, the authors also describe a diminished degree of coupling of HPC (but not mPFC) gamma to the SO phase following RE inhibition, suggesting that the opposite of their conclusion may be the case (i.e., the off-line, slow-wave pathway operates to synchronize activity in a mPFC-to-HPC-dependent manner). One point to highlight in this regard that may be relevant to the results reported by Ferraris et al. (2018) during anesthesia is that they evoked SO activity by supplementing urethane anesthesia with ketamine-xylazine. Ketamine–xylazine anesthesia is known to inflate SO-related coherence between neocortex and hippocampus compared with urethane alone (Sharma et al., 2010). In addition, ketamine–xylazine anesthesia is also known to alter the dynamics of SO-related neocortical unit activity (Chauvette et al., 2011) compared with natural sleep. Finally, ketamine–xylazine anesthesia inflates SO-related gamma activity in the forebrain (Hong et al., 2010; Shaw et al., 2015; Furth et al., 2017), but also specifically in the hippocampus compared directly with urethane anesthesia (Sharma et al., 2010). In this respect, we feel that urethane-alone experiments more closely resemble natural sleep.

Slow-wave sleep and, indeed, the SO itself have been implicated in off-line memory consolidation (Steriade and Timofeev, 2003; Stickgold, 2005; Dickson, 2010). Transcranial stimulation at SO frequencies enhanced the retention of declarative memories in a human population (Marshall et al., 2006). Slow-wave sleep appears to be especially well suited for facilitating spike timing-dependent plasticity processes that are critical for memory formation (González-Rueda et al., 2018). Moreover, the negative trough of the SO is ideally suited for optimizing synaptic plasticity within local circuits (Mölle et al., 2011; Niethard et al., 2018). Synaptic excitability in general is enhanced during SO (compared with theta) states, but this is especially prominent during the falling phase (positive to negative) of the SO (Schall et al., 2008). This is the precise timing we found that RE units were phase coupled to, with respect to the mPFC SO. The SO, as such, represents an excellent platform for coordinating communication across large and disparate cortical sites (Buzsáki, 1996, 1998; Steriade and Timofeev, 2003; Dickson, 2010; Cox et al., 2014), and the RE is an ideal mediator for exactly this activity pattern.

### Conclusion

Here we show that selective inhibition of the thalamic RE impairs slow oscillatory coordination between neocortical and hippocampal sites. We first demonstrated that RE neurons were strongly and rhythmically coupled to the negative phase of the neocortical SO. Stimulating either directly at the level of the RE or its axonal projection bundle, or even at the level of the mPFC, reliably produced an evoked HPC potential maximal at the level of SLM. Chemogenetic inhibition of the RE abolished the HPC potential evoked by mPFC stimulation, demonstrating that we could functionally impair this disynaptic circuit. Doing so caused a robust decrease in mPFC–HPC synchronization at SO frequencies. Together, our data demonstrate that the RE has a critical role in mediating frontal cortical–HPC coordination, particularly during slow-wave/off-line states. This has marked implications for sleep-dependent memory consolidation and highlights the RE as an exciting avenue for future study.

## Synthesis

Reviewing Editor: Menno Witter, Norwegian University of Science and Technology Decisions are customarily a result of the Reviewing Editor and the peer reviewers coming together and discussing their recommendations until a consensus is reached. When revisions are invited, a fact-based synthesis statement explaining their decision and outlining what is needed to prepare a revision will be listed below. The following reviewer(s) agreed to reveal their identity: NONE. Note: If this manuscript was transferred from JNeurosci and a decision was made to accept the manuscript without peer review, a brief statement to this effect will instead be what is listed below.

This transferred manuscript is a revised version based on two through evaluations of the original manuscript. The authors have adequately responded to the comments and questions raised in the two original evaluations. The paper has improved substantially and provides partially confirmatory and partially new data contributing to our understanding of the interplay between the mPFC and the hippocampus, and in particular the role played by RE in synchronized slow oscillatory activity, which may be fundamental for slow-wave sleep-related episodic memory consolidation.
